# A Christmas Opportunity

**Published:** 1920-12-18

**Authors:** 


### The Hospital

The Workers' Newspaper of Administrative Medicine and Institutional Life, Administration, National Insurance and Health.

No. 1802, Vol. LXIX. SATURDAY, DECEMBER 18, 1920. PRICE TWOPENCE

### A CHRISTMAS OPPORTUNITY.

In what mood, we wonder, will our readers turn this year to the summary list of hospital requirements which we publish, as usual, before Christmas? (See p. 275.) At all events, we give the facts straightforwardly, for each institution, so that those who may have been dazed by the alarming statements of the newspapers may understand the basis of these fears. But, however serious the position of certain hospitals may be, Christmas is not the time for dismal thoughts, nor do we think that the charitable public, who reads these pages, will read them with a guilty conscience. For the truth is not that the support of voluntary hospitals has diminished, but that the purchasing power of money has declined, so that more than £2 has to be spent where £1 was sufficient before the war. The voluntary gifts have grown, but they have not grown so fast as prices. The consequence has been that the ordinary appeals for money, reasonably well though they have been met, still prove insufficient to support the institutions. These have, therefore, been obliged to find fresh sources of income by imposing a charge toward their maintenance on all patients who are able to pay, and, in industrial areas, by asking the working men to increase their weekly contributions. In almost every case these two plans have been in some degree successful, but still the expenditure runs ahead of the available income. Consequently, a novel appeal has to be made to those who believe in the voluntary system, an appeal to their heads as well as to their hearts, to their intelligence as well as to their purses.

Every reader into whose hands this issue comes has his own calling and place of business. We therefore venture to ask him, What is the spirit of that place in regard to hospitals? Does it know the value of these institutions; does it give a collective thought to the centres from which its doctors and nurses come, and to the needs of the sick to which they minister? If not, his opportunity is plain. As one who is already interested in the voluntary hospitals, and who believes that the best thanksgiving for health is to do something for those who are ill, the reader should ask his colleagues, wherever he works, to start a joint collection, and hand its weekly proceeds to the nearest hospital.

The vast sums of money needed can be raised, as all very large sums nowadays are raised, by a multitude of quite small but regular contributions. The profits of the reader's own business are made in this fashion, and the incomes of hospitals increasingly spring from a similar source. If every office, place of business, shop, and factory will start a weekly collection of twopence a head, supplemented by a similar total from the head of the firm, the London hospitals will have the money which they need. What many of the large factories in London are doing by affiliation to the Hospital Saturday Fund, the offices and shops have begun to do. But there must be no exceptions, if the present difficulties are to be met. We therefore ask each reader not so much what he can give himself, as what his place of business can give by a small weekly contribution. A little public spirit can do more this Christmas than any private purse. We do not ask the reader to turn out the contents of his pocket, but to turn over this matter in his mind. What he has done in the past by giving his office also can do. We ask him to start a weekly collection there. If he needs advice in this matter he will find ready help in Mr. Philip Inman, the Secretary of the Metropolitan Hospital Saturday Fund, who has already offered to make things simple for each of the new centres which have happily started this plan in recent weeks. Some one in each office must make the original suggestion, and who can that be but the man who already knows what the voluntary system means? He will find an apt argument in the contrast which the voluntary hospitals provide to the State machinery lately extended by the provisions for unemployment insurance. The delays, red tape, forms, and interference make State benefits almost worse than the ills which they were designed to meet. For the recipient of State help ceases to be a free man. He becomes the victim of a department, badgered and annoyed at every turn—a number, not a human being. From all this the voluntary system saves us. It is elastic, humane, considerate. It is not tied and bound by regulations. It can meet exceptional cases; it is free to strain a point. These are the virtues of the voluntary system; but the system can only bear the fruits of freedom so long as people voluntarily provide it with funds. To preserve the virtues of voluntaryism voluntary givers must abound. The one is inseparable from the other.

A little voluntary enterprise is all that is required, and by the New Year thousands of places of business in London will be having their weekly collections. It all depends on the willingness of our readers to give a little personal service, to set an example, to become missioners of this plan. It is not as if there were any opposition. The difficulty resides in the reader, not in the course proposed to him here.

